# Prisoners of Solitude: Bringing History to Bear on Prison Health Policy

**DOI:** 10.1016/j.endeavour.2016.07.001

**Published:** 2016-09

**Authors:** Margaret Charleroy, Hilary Marland

**Affiliations:** Centre for the History of Medicine, Department of History, University of Warwick, Humanities Building, Room 449, Coventry, CV4 7AL, United Kingdom

## Abstract

• Importance of history for prison health policy.
• Historical origins of solitary confinement.
• Debates over health effects of solitary confinement.

Season two of the popular prison drama *Orange is the New Black* opens in a small concrete cell, no larger than a parking space. The cell is windowless and sparsely furnished; it holds a toilet, a sink and a limp bed. The only distinguishing feature we see is a mural of smeared egg, made by the cell's resident, the show's protagonist Piper Chapman. When a correctional officer arrives at this solitary confinement cell, he wakes her, and mocks her egg fresco. “This is art,” she insists. “This is a yellow warbler drinking out of a daffodil.” Her rambling suggests the confusion and disorientation associated with inmates in solitary confinement, who often become dazed after only a few days in isolation. As the scene continues, we see Piper exhibit further symptoms associated with both short- and long-term solitary confinement—memory loss, inability to reason, mood swings, anxiety—all indicating mental deterioration and impaired mental health. In this and other episodes, we begin to see solitary confinement as the greatest villain in the show, more villainous than any character a writer could create. The new and growing trend of television prison dramas like *Orange is the New Black* brings the issue of solitary confinement, along with other issues related to incarceration, to a more general audience, exposing very real problems in the failing contemporary prison system, not just in America, but worldwide. The show's success leads us to ask how history, alongside fictional dramas and contemporary case reports, can draw attention to the issue of solitary confinement.

Solitary confinement harms prisoners who were not mentally ill upon entry to the prison and worsens the mental health of those who were. Both historical and contemporary evidence has demonstrated how both short- and long-term solitary confinement threatens the physical and mental health of those who endure it. So how and why has it become one of the most widely used means to control and punish inmates in the Western prison system, one involving around 80,000 people in prison currently in the US? And, how can historical perspectives inform contemporary discussions concerning the effects of solitary confinement on the mental health of inmates?

The health effects of solitary confinement are currently being debated by policymakers, governments, academics, prison staff, criminologists, psychiatrists and historians on both sides of the Atlantic. The potency of historical evidence—on this and other themes related to mental health and the criminal justice system—was on display at a recent workshop in London on “The Prison and Mental Health,” co-convened by Professor Hilary Marland at the University of Warwick, England and Dr. Catherine Cox, based at University College Dublin, Ireland. The event involved historians, criminologists, psychiatrists working in prison settings, representatives of prison reform organizations and policymakers, who came together to explore the potential of history to inform, enhance, and shape current debates on the prison and mental health. The event, showed, above all, how a historical perspective allows us to link contemporary debates around solitary confinement with the prison regimes and their associated philosophies of rehabilitation, treatment and punishment that inspired this lingering practice. It also underlined the close and enduring relationship between solitary confinement and high rates of mental illness. Until now, a historical perspective has remained largely absent from academic and legal writing on a topic that strives to produce policy changes in prisons. Yet history can make a powerful contribution to these discussions, documenting shifts in prison policy and discipline and acting as the wellspring of narratives that highlight the devastating impact of solitary confinement over the longue durée. Viewing contemporary policy through a historical narrative exposes sources of enduring problems, as well as giving them faces, names and stories.

In the past decade, prison administrators in both the United States and England have significantly increased the use of solitary confinement as a means of “managing” difficult prisoners. But solitary confinement, as illuminated at the workshop, is far from new. Its roots can be traced to the rise of the modern penitentiary in the early nineteenth century, when isolating all inmates was used as a means of rehabilitation, or so prison reformers and administrators thought. What began as a program to rehabilitate inmates in America during the early nineteenth century, and was brought to England just over a decade later, in practice led to increased rates of mental illness among prisoners, which the prison physicians and chaplains recorded. A nineteenth-century inmate at Eastern State Penitentiary echoed the experience of Piper: “In the gloomy solitude of a sullen cell there is not one redeeming principle. There is but one step between the prisoner and insanity.”^1^

Despite these effects, solitary confinement sprang from high-minded motives. At the start of the nineteenth century, prison reformers reconsidered the relationship between punishment and reformation, and experimented with prison regimes and architecture. In 1787, a coalition of Philadelphia social reformers, mostly Quakers, and led by Benjamin Rush, formed the Philadelphia Society for Alleviating the Miseries of Public Prisons. In direct contrast to the corporal and capital punishment employed in existing prisons, the Pennsylvania reformers believed that, once isolated, prisoners would be reformed through silent, spiritual reflection. To achieve these reformative goals, they designed a prison where inmates would have little or no contact with either other prisoners or staff. This strict isolation, it was hoped, would allow inmates to reflect upon their actions, inducing penitence and promoting deep-seated moral and spiritual reform.

These reforms were the foundation of what became known as the Pennsylvania system—also known as the separate system—of prison policy and inmate reform. The system was first implemented at Eastern State Penitentiary in Philadelphia, Pennsylvania in 1829. With the construction of a new prison, advocates of the Pennsylvania system were able to build the assumption of solitary confinement into the very architecture of the prison in a way that had never before been attempted. Prisoners ate all meals in their cells. Cell walls were thick and prevented inmates from communicating with one another. Attached to each cell was a small yard for private exercise by inmates. The need for these solitary cells guided the physical design of the prison and led to the famed radial design, pioneered by John Haviland (Fig. 1).

Jeremy Bentham's panopticon—though never actually built—was the inspiration for Haviland's radial plan. At the center of Haviland's structure stood an eighty-foot tower, which served as a viewing platform for prison guards who would thus be able to observe all of the prison corridors from a single vantage point and monitor inmate behavior at all times. Seven single story wings radiated from the central tower. The tower guards could see the prisoners in their individual exercise yards, though the prisoners themselves would have had no contact with one another because inmates were given time in their individual exercise yards at staggered times throughout the day to diminish the possibility that they would communicate with one other. Indeed, communication between prisoners was punished harshly. Eastern State was a penitentiary in a literal sense. The physical structure, which reinforced strict solitude, was designed to encourage introspection and, ultimately, penitence. Haviland's radial design for Eastern State Penitentiary became the most widely copied prison format in the nineteenth-century United States.

Less than a decade after Eastern State Penitentiary opened its doors, it became apparent that isolation was causing mental breakdown amongst the prisoners. Reports describing the effects of the Pennsylvania system on the minds of inmates appeared in annual reports of the Prison Discipline Society, *The Journal of Prison Discipline and Philanthropy*, and numerous other publications popular among social reformers and scholars. In the 1838 report of the Prison Discipline Society, the “Effects of the System of Solitary Confinement, Day and Night, on the Mind” was included as subcategory of discussion, one that was retained through the following decade.^2^ Their argument was simple: isolation produced higher rates of mortality and insanity among prison inmates.

English prison reformers visited American prisons at the height of debates about the merits and drawbacks of solitary confinement. In 1833 William Crawford, founder member of the Society for the Improvement of Prison Discipline, was commissioned by the British government to report on American prisons and penal ideas. He returned to England entranced by the system in operation at Eastern State Penitentiary, eager to apply the same model of prison discipline in the new prison being planned in London, Pentonville Model Prison.

Crawford and Reverend Whitworth Russell, who were appointed prison inspectors for London in 1835, were vigorous advocates of the separate system and brushed off warnings of the dangers inherent in the regime to the mental state of the prisoners that American reformers put forth. They argued that what distinguished their model at Pentonville from the Philadelphia system was the access prisoners would have at all times to the prison officers, notably the chaplains. Pentonville's critics were not convinced. During his travels in America, author Charles Dickens most wanted to see two sights: the falls at Niagara and Eastern State Penitentiary. His visit to Eastern State prompted a critical response. In particular, he condemned the system of solitary confinement imposed there in his *American Notes*, published in 1842, the year Pentonville took in its first prisoners. Encountering several of Eastern State's prisoners, he referred to how one was “a dejected heart-broken wretched creature,” another “a helpless, crushed, and broken man.”^3^ Dickens concluded, “I hold this slow and daily tampering with the mysteries of the brain to be immeasurably worse than any torture of the body.”^4^ An editorial in the London *Times*, which campaigned against the separate system, predicated that insanity would be a “probable,” even “inevitable,” outcome of the Pentonville regime.^5^

Pentonville Model Prison heralded the launch of a new prison system and approach to punishment in Britain when it opened in 1842. Like Eastern State Penitentiary, Pentonville was intended, through religious exhortation, rigorous discipline, moral training and the imposition of separation in its most extreme form, to produce true and deep repentance and rehabilitation in its convict population. The approach was exacting and rigorous. Pentonville, with its 500 inmates housed in tiered lines of cells radiating from a central block, operated like a machine, with every minute of a convict's day, from the first bell at 5:30 a.m. until lights out at 9:00 p.m., regimented, directed and observed in meticulous detail. Prisoners were forbidden to communicate with each other, and locked twenty-three hours a day isolated in their cells, where they ate, worked and slept. As at Eastern State, inmates were moved through the prison with their faces covered by hoods, seated in chapel in separate stalls and exercised in separate airing yards.^6^

Cracks in the system quickly appeared and were recorded in the journals compiled by prison medical officers and chaplains, the latter particularly staunch advocates of the separate system and key figures in its implementation. Within weeks of its opening, Pentonville was racked by alarming cases of mental breakdown, delusions, hallucinations, panic, depression, anxiety and morbid feelings, according to medical staff and chaplains. Prisoners declared that they were visited by the spirits of the dead, that they were being poisoned, that there were snakes coiled around the bars of their cells and that “things” crawled out of the ventilation system. The chaplains and medical officers were preoccupied on a daily basis with attempts to subdue and calm prisoners intent on violence, suicide, or self-harm. Official reports, with some reluctance, confirmed the relationship between high levels of mental disease and the rigor with which the separate system was implemented. As a result, already by the mid-nineteenth century, the separate stalls were dismantled in the chapel, solitary exercise and the wearing of masks discontinued and the period spent in solitary confinement reduced from eighteen to twelve and then to nine months by 1853. However, this moderated separate system endured in Britain for the remainder of the nineteenth century, driven in the last quarter of the century by ideas of appropriate punishment rather than reform. It continued to be associated with the mental breakdown of Britain's growing prison population (see Fig. 2).^7^

This system of physical isolation was expensive and cumbersome, and increasingly controversial. Even at Eastern State, where it was created, it gradually broke down. Most prisons built in the United States in the nineteenth century were products of the new philosophy of the Auburn system, which required that prisoners work in association—and in silence—during the day and sleep in solitary cells at night. Although the Pennsylvania system endured in Europe, South America and Asia, by the opening decades of the twentieth century, the United States had largely abandoned it and Britain had reduced the use of solitary confinement, hastened by the widespread, and now acknowledged, mental health problems related to the isolation of inmates.

A practice designed as a means to rehabilitate inmates under the regime of the Pennsylvania system, and abandoned for its abject failure to do so, would be revived in the late-twentieth century as a tool of punishment. The separate system was in essence solitary confinement, albeit one that involved all prisoners and that was associated, particularly in its early years, with reform and rehabilitation rather than punishment. Even under the separate system, prisons superimposed isolation in dark cells as a form of punishment for disruptive behavior, for disobeying prison rules or for feigning mental illness. Pentonville Prisoner no. 683, for example, was given three days in the dark cell with a punishment diet in June 1845 for refusing to work and attempting to create “a belief that he is an imbecile.”^8^

Today, solitary confinement is also used as a punishment and can be envisaged as a form of prison within a prison. It is used more widely in the United States, with the population of individuals confined in solitary confinement equaling nearly the entire prison population of the United Kingdom, where fewer than 500 inmates are estimated to be in solitary confinement at any given time (a modern solitary confinement cell is shown in Fig. 3).^9^ As Suzie Nielson, former inmate, describes it: “While there is no universally agreed-upon definition, modern solitary—also called supermax, isolated segregation, and “the box”—is commonly understood to involve confinement to a small cell for 22 to 24 hours a day.”^10^ Under such regimes, prisoners are denied access to leisure activities and hobbies, and, just as in the nineteenth century, are forbidden from communicating with other prisoners. They are often handcuffed and shackled on the rare occasions when they leave their cells. Not all prisoners are sent to segregation units, as they are officially designated, as a form of punishment. Some, as Erwin James explained in a recent *Guardian* article, “engineer” their move, seeking respite from life on the chaotic main wings of the prison, to escape risks of violence from other prisoners, or to gain easier access to prison managers.^11^

Contemporary studies on the health effects of solitary confinement conclude, in line with the observations of nineteenth-century reformers on both sides of the Atlantic, that long-term isolation can cause hallucinations, panic attacks, impulse control, paranoia, anxiety, confusion, obsessions and memory loss. *Deep Custody*, a report produced by the English Prison Reform Trust in 2015, and also discussed at the recent London workshop, highlighted the “toxic” effects of segregation, caused by “social isolation, reduced sensory input/enforced idleness and increased control of prisoners even more than is usual in the prison setting.”^12^ Over half of the sixty-three individuals interviewed reported that they had three or more of the following symptoms after forced isolation: anger, anxiety, insomnia, depression, difficulty in concentration and self-harm.^13^ In the words of one of the prisoners interviewed: “The longer you’re here, the more you develop disorders. Being in such a small space has such an effect in decreasing your social skills. It looks rosy, but it has such a negative effect. It's isolation to an extreme.”^14^

Recent studies also note high rates of self-mutilation and suicide among inmates in solitary confinement. One 1995 study found that prisoners in solitary confinement accounted for nearly half of all suicides in California's prisons between 1999 to 2004.^15^ Nor are the negative effects of isolation limited to prisoners’ time in segregation. Those who are released from solitary confinement into the general population of the prison often have difficulties adjusting due to social anxiety and social atrophy from prolonged isolation. Prisoners often report bizarre and disturbing subjective experiences after they leave isolation. Neilson writes: “Some say the world regularly collapses in on itself. Others report they are unable to lead ordinary conversations, or think clearly for any length of time.”^16^

This is changing. In 2011, hunger strikes by inmates in California's prison ended when the system agreed to provide calendars to inmates in long-term isolation (Fig. 4). In September 2015, the state of California announced plans to overhaul the use of solitary confinement in the state's prisons. The agreement came after a lawsuit was filed against the state by inmates held in isolation for ten or more years at California's Pelican Bay Prison. Under the provisions of the settlement, clearer guidelines for use and time of isolation were laid out; prisoners can no longer be kept in isolation indefinitely and inmates cannot be isolated because of gang affiliation.

Other states in America, like New York, are piloting alternatives to solitary confinement in their Clinical Alternatives to Punitive Segregation (CAPS) program, launched in 2013. Inmates assigned to these units are not locked in isolation, but instead are “locked out” of their cells, encouraging them to participate in therapeutic activities, including psychotherapy, art, educational programs and mental health counseling (both individual and group settings) during the daytime. Although the cost of these units has limited their adoption in the state-wide prison system, prisons that do offer this alternative to solitary confinement report phenomenal success, measured by a reduction in self-harm, suicide and hospitalization.

However, the speed of change is slow and uneven, as illuminated by the 2015 report *Deep Custody*, referred to above. Though in comparison to the US, the scale of solitary confinement is much smaller in the UK—in January 2015, the total segregation capacity in England and Wales was 1,586 cells, while close supervision centers had a capacity of just 54—many prisoners still end up in cellular confinement for long periods, as result of poor provision rather than as punishment for infringement of rules or a perceived need for segregation.^17^ Though prison reform is high on the agenda of the current British government, and welcomed by prison reform organizations, there is little evidence to suggest that the problem of prisoners being locked in their cells for excessive periods is being tackled in an environment of staff shortages and very poor conditions in decaying structures dating from the Victorian period. A recent report on Wormwood Scrubs Prison in London revealed that many prisoners had less than two hours a day “unlocked” and all had only forty minutes of outdoor exercise a day, less than the time prescribed at Pentonville in 1842.^18^

As described in the opening vignette, an inmate's experience in solitary confinement is shown vividly in episodes of the television series *Orange is the New Black* to an audience who likely will never face the deleterious effects of isolation. The series is based on the book *Orange is the New Black: My Year in a Women's Prison*, by Piper Kerman, who was herself an inmate at a United States Federal Correctional Facility for thirteen months. Since the publication of her memoir and production of the series, Piper Kerman has become a vocal advocate on behalf of incarcerated individuals. In June 2015, Kerman testified before the United States Senate Judiciary Subcommittee hearings on solitary confinement. Her personal experience gave her authority. But it was the transformation of her personal story into a widely consumed television narrative that gave her influence. And it gave a voice to many individuals currently isolated in solitary confinement.

Historical research too has a role to play in arguing for the amelioration of solitary confinement, contributing to the same debates and work that Kerman and others are doing. Whatever form it took and whether driven by reformist principles, punishment, convenience, or prisoner requests for segregation, history can demonstrate the devastating consequences of separate confinement on prisoners and in particular their mental wellbeing, establishing connections and continuities over two centuries. History adds significantly to the weight of evidence and force of argument on the destructive impact of isolation and joins forces with the reports of policymakers and prison reform organizations in urging that new approaches must be sought and the impact of solitary confinement mitigated.

## Figures and Tables

**Fig. 1:**
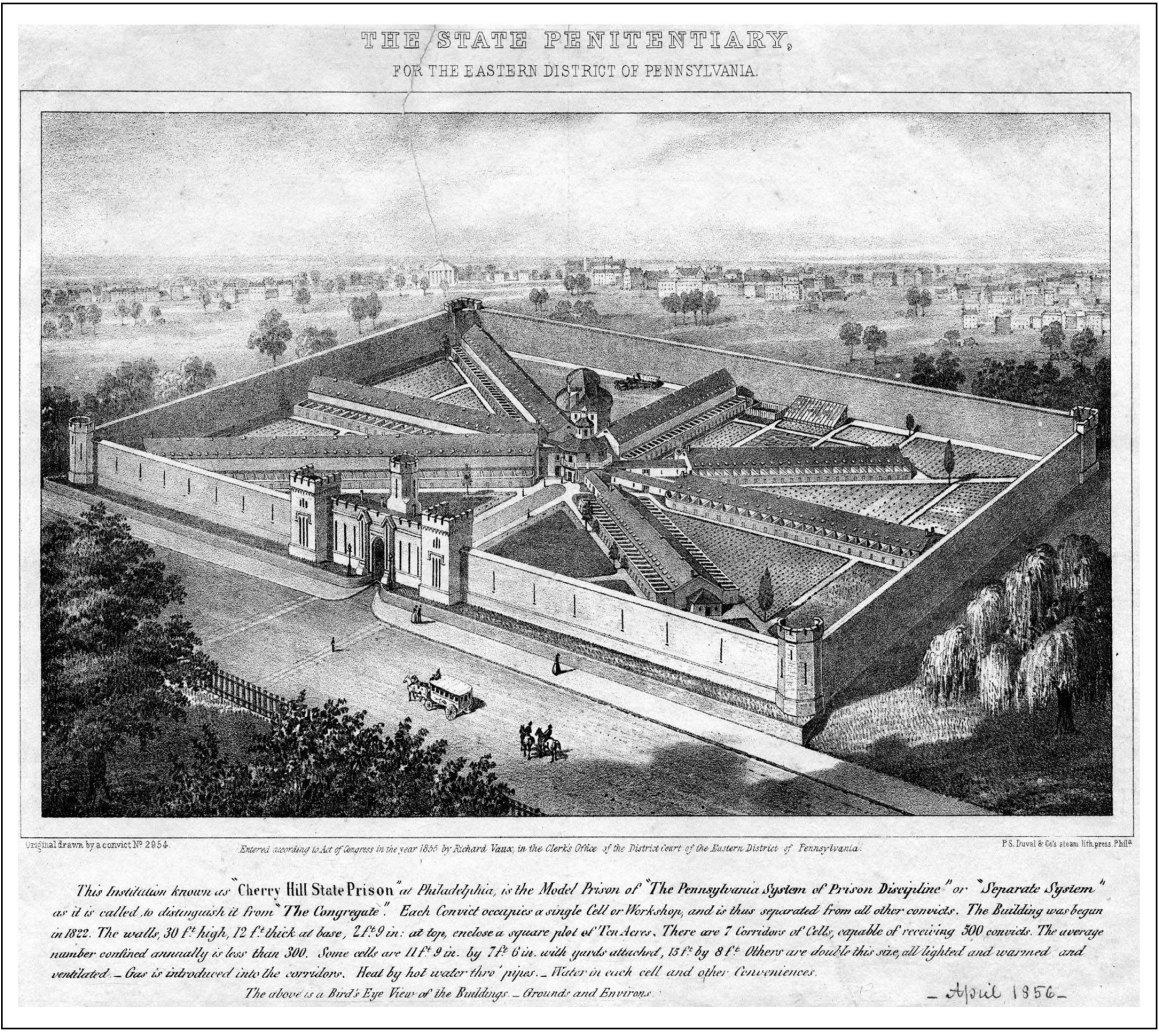
Radial design of the Eastern State Penitentiary, designed by John Haviland, as it looked in 1855. The lithograph was made by Samuel Cowperthwaite, an artist and convict number 2954 at the penitentiary. The caption reads: “This Institution known as ‘Cherry Hill State Prison’ at Philadelphia, is the model prison of ‘The Pennsylvania System of Prison Discipline’ or ‘Separate System’ as it is called to distinguish it from ‘The Congregate.’ Each Convict occupies a single Cell or Workshop, and is thus separated from all other convicts. The Building was begun in 1822. The walls, 30 ft. high, 12 ft. thick at base, 2 ft. 9 in. at top, enclose a square plot of Ten Acres. There are 7 Corridors of Cells, capable of receiving 500 convicts. The average number contained annually is less than 300. Some cells are 11 ft. 9 in. by 7 ft. 6 in. with yards attached, 15 ft. by 8 ft. Others are double this size, all lighted and warmed and ventilated.—Gas is introduced into the corridor. Heat by hot water thro’ pipes.—Water in each cell and other Conveniences. The above is a Bird's Eye View of the Buildings—Grounds and Environs.”

*Credit:* Library Company of Philadelphia, used with permission.

**Fig. 2:**
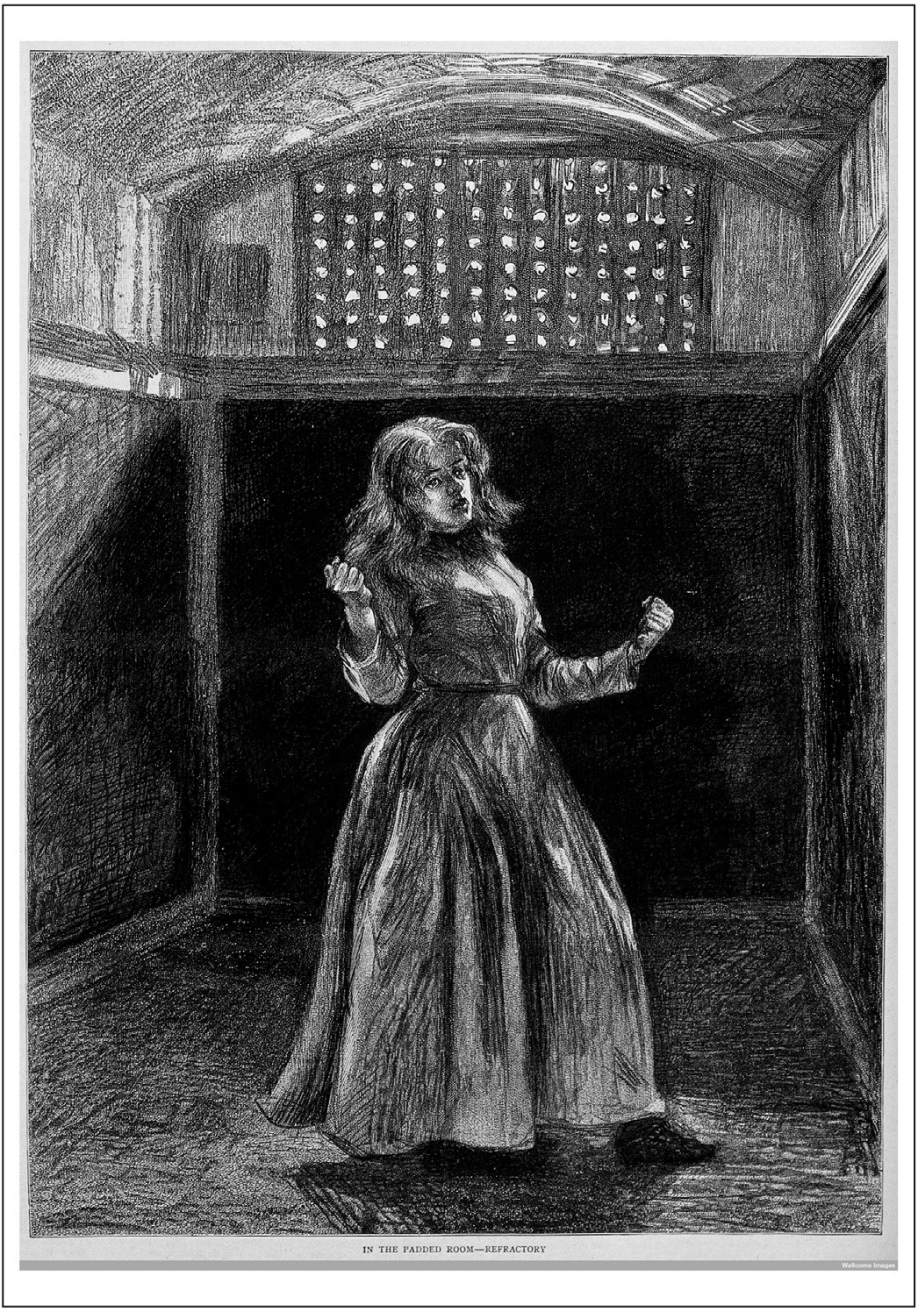
A woman prisoner in solitary confinement at Woking Prison, England.

*Credit:* Wellcome Images, used with permission.

**Fig. 3:**
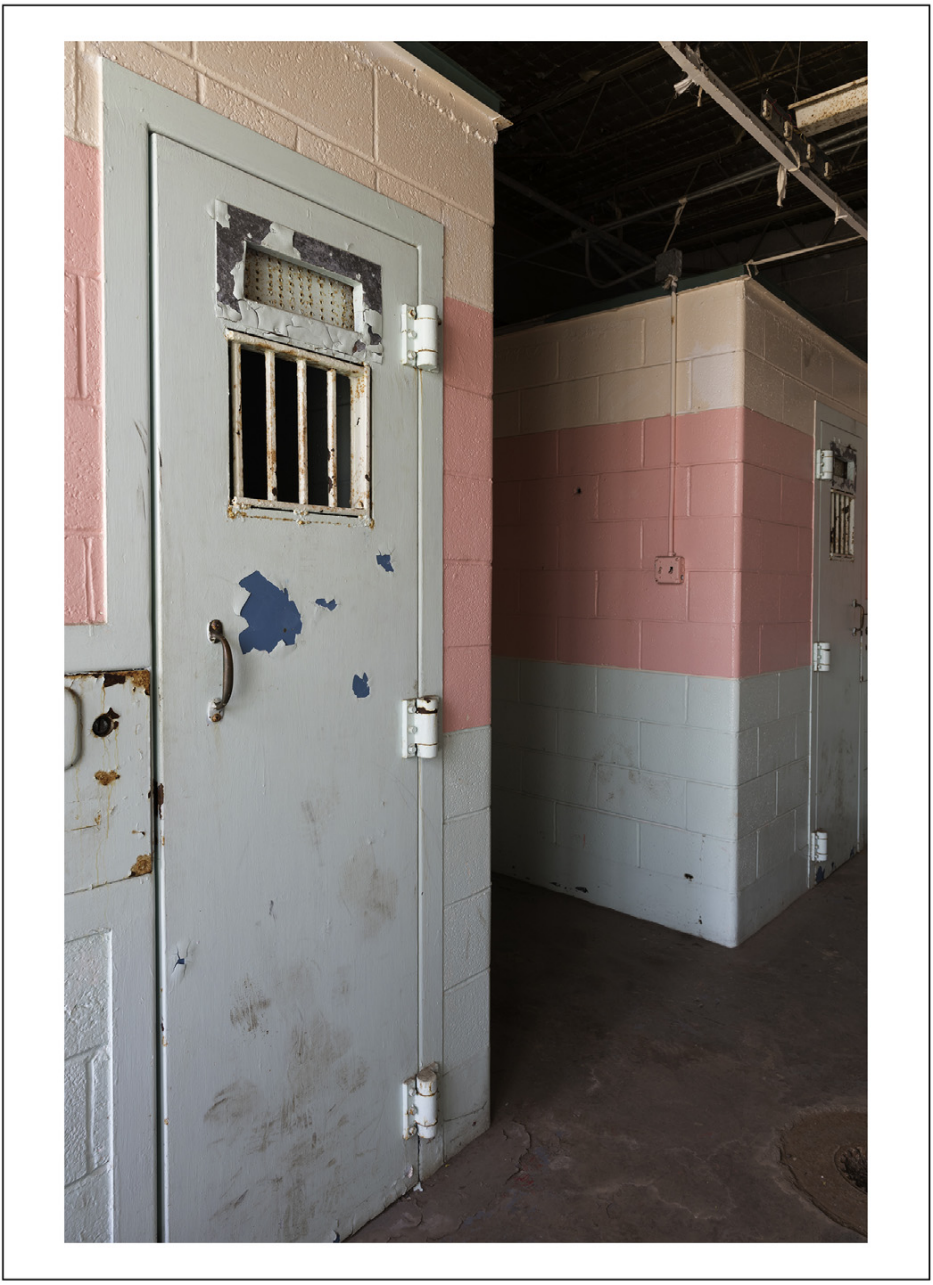
Solitary confinement cells at the West Virginia State Penitentiary, a retired, gothic-style prison in Moundsville, West Virginia, that operated from 1876 to 1995.

*Credit:* Library of Congress.

**Fig. 4:**
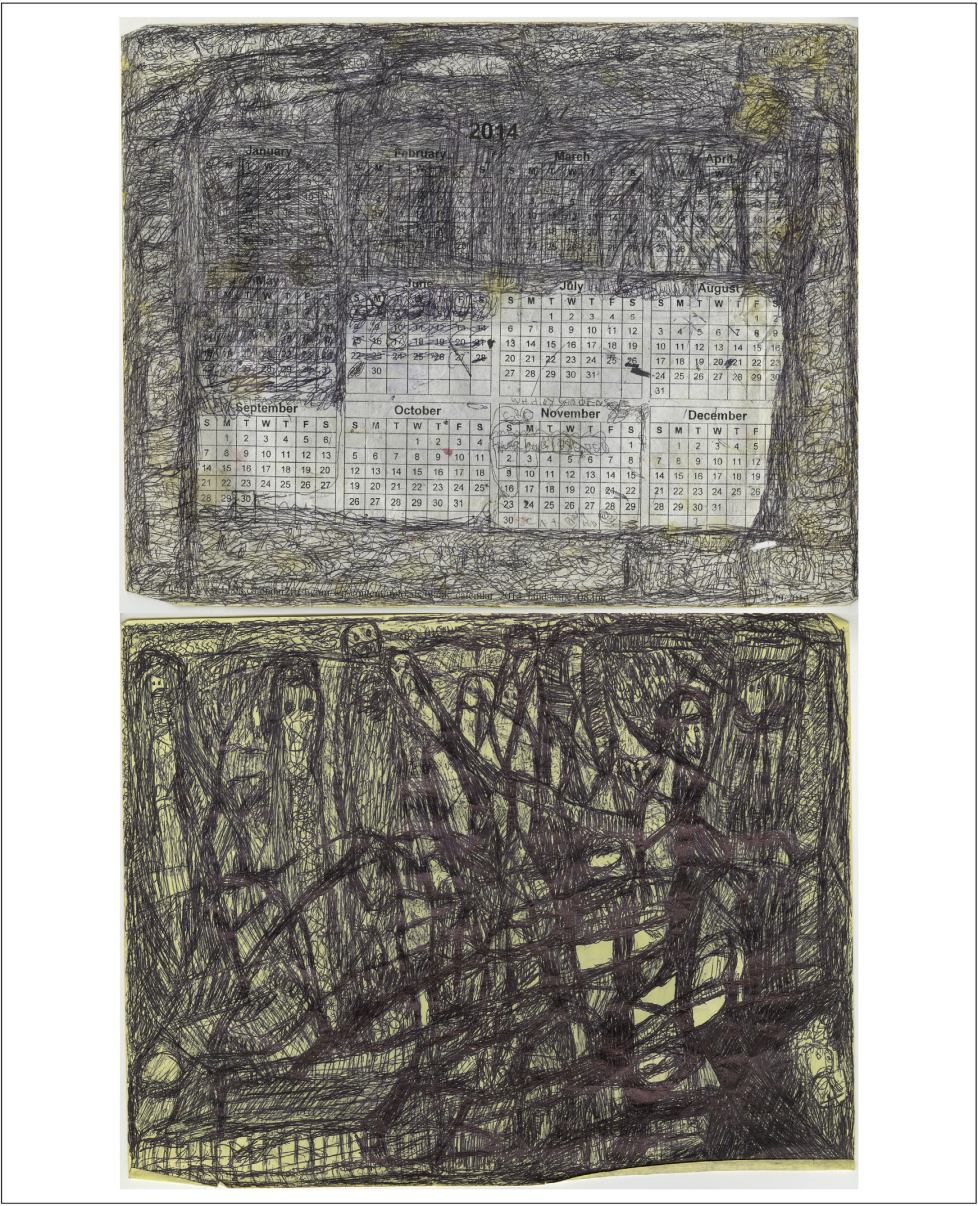
Drawings of one-time California prisoner Ernest Jerome DeFrance. DeFrance made these images while incarcerated in the California prison system, where he spent extended periods of time in solitary confinement. He submitted these works to *Sentenced: Architecture and Human Rights*, an exhibition held at the University of California, Berkeley in fall 2014, produced by Architects, Designer and Planners for Social Responsibility (ADPSR). These works by Ernest Jerome DeFrance were later featured in the show *Demos: Wapato Correctional Facility* by artist collective ERNEST at c3:initiative in Portland, Oregon in September 2015.

*Credit:* Ernest Jerome DeFrance.

## Footnotes

1 State Penitentiary for the Eastern District of Pennsylvania Records, Series 4, Prisoners’ Correspondence, Prisoners’ Letters, American Philosophical Society, Philadelphia, PA.

2 *Thirteenth Annual Report of the Board of Managers of the Prison Discipline Society* (Boston: The Society's Room, 1838), 236.

3 Cited in David Wilson, “Testing a Civilisation: Charles Dickens on the American Penitentiary System,” *The Howard Journal of Criminal Justice* 48 (2009): 280–96, on 290–91.

4 Charles Dickens, *American Notes for General Circulation: Vol 1* (London: Chapman and Hall, 1842), 239.

5 Editorial, *The Times*, May 1, 1841.

6 See Michael Ignatieff, *A Just Measure of Pain: The Penitentiary in the Industrial Revolution 1750–1850* (New York: Pantheon, 1978), 3–8; *Second Report of the Commissioners for the Government of Pentonville Prison* (London: Clowes and Sons, 1844), 18–20.

7 Pentonville has been explored in more detail in Catherine Cox and Hilary Marland, ““He must die or go mad in this place”: Prisoners, Insanity and the Pentonville Model Prison Experiment, 1842–1852,” *Bulletin of the History of Medicine*, forthcoming 2017.

8 Pentonville Minute Book 1845–46, 7 June 1845, The National Archive, London, UK.

9 Elisa Moser, “Solitary Confinement in Great Britain: Still Harsh, But Rare,” *Solitary Watch* (January 19, 2012), http://solitarywatch.com/2012/01/19/solitary-confinement-in-great-britain-still-harsh-but-rare/.

10 Suzie Neilson, “How to Survive Solitary Confinement: An Ex-Convict on How to Set Your Mind Free,” *Nautilus* 32 (January 28, 2015). http://nautil.us/issue/32/space/how-to-survive-solitary-confinement.

11 Erwin James, “Prison Segregation Units are a Breeding Ground for Mental Health Problem,” *The Guardian*, December 17, 2015, http://www.theguardian.com/commentisfree/2015/dec/17/mental-health-prison-segregation-units-prisoners.

12 Ibid.

13 Sharon Shalev and Kimmett Edgar, *Deep Custody: Segregation Units and Close Supervision Centres in England and Wales* (London: Conquest Litho, 2015), 93.

14 Ibid., 55.

15 Ian Lovett, “California Agrees to Overhaul Use of Solitary Confinement,” *New York Times*, September 1, 2015, http://www.nytimes.com/2015/09/02/us/solitary-confinement-california-prisons.html.

16 Neilson, “How to Survive Solitary Confinement” (ref. 10).

17 Shalev and Edgar, *Deep Custody* (ref. 13), 5.

18 Report on an Announced Inspection of HMP Wormwood Scrubs by HM Chief Inspector of Prisons, November 30–December 4, 2015, https://www.justiceinspectorates.gov.uk/hmiprisons/inspections/hmp-wormwood-scrubs-2/.

